# Laboratory Automation in the Microbiology Laboratory: an Ongoing Journey, Not a Tale?

**DOI:** 10.1128/JCM.02592-20

**Published:** 2021-02-18

**Authors:** Stefan Zimmermann

**Affiliations:** a Department of Infectious Diseases, University Hospital Heidelberg, Heidelberg, Germany; NorthShore University HealthSystem

## Abstract

Clinical chemistry laboratories implemented fully automated devices decades before microbiologists started their subtle approaches to follow. Meanwhile several papers have been published about reduced time to reports, faster workflows, and increased sensitivity as results of lab automation. While the journey of automating microbiology workflows step by step was fascinating and beneficial, monetary aspects were uncommon in most publications. In this issue of the *Journal of Clinical Microbiology*, K. Culbreath, H. Piwonka, J. Korver, and M. Noorbakhsh (J Clin Microbiol 59:e01969-20, https://doi.org/10.1128/JCM.01969-20) calculate the benefits of total lab automation in terms of cost savings and lab efficiency in a “tale of four laboratories.” The authors here provide facts and solid calculations about the benefits achieved in four different-sized labs after implementation of full laboratory automation.

## Abstract (precis)

Clinical chemistry laboratories implemented fully automated devices decades before microbiologists started their subtle approaches to follow. Meanwhile several papers have been published about reduced time to reports, faster workflows, and increased sensitivity as results of lab automation.

## TEXT

Although clinical chemistry laboratories had a huge advantage in total automation of their workflows in many, yet not all, analysis steps, microbiology had some modest approaches to customize specific devices. Automated blood culture systems and instruments for identification and antimicrobial susceptibility testing (ID/AST) were introduced more than 30 years ago. The major backbone of the workload in the lab, specimen handling, plate inoculation, incubation, and reading remained manual processes.

About 10 years ago, devices came to the market that allowed specimen inoculation and, later, also plate transportation to smart incubators, which then were able to read plates digitally (1). This development was accompanied by numerous new technological approaches (2), but only a few of them really became routine procedures. The idea of a completely automated microbiology workflow beginning with the arrival of specimens in the lab to the final reporting in the lab information system (LIS) grew in the field of clinical microbiology and evolved rapidly (3).

Today, two major companies, BD Kiestra (USA) and Copan (Italy, meanwhile supported also by bioMérieux) offer complete laboratory automation systems for microbiology labs. The decision to use either the one or the other is a difficult process, which depends on many variables in the lab, e.g., space, specimen types, or connectivity to already installed instruments (4). Both systems have proven to show multifaceted benefits for the laboratory and the hospital. Numerous processing steps could be eliminated from the workflow cascade, e.g., transport of plates to inoculation area, incubator, or reading desks or labeling and streaking of plates.

As a major advantage, the systems provide more rapid results and significantly reduced the time to report. While manually processed urine specimens, at least in our lab, were read on day 1 and day 2 after inoculation, the incubation time could be reduced in the automated workflow to 20 h. Other labs were even more successful and could reduce the turnaround time (TAT) to 16 h (5). Patients suffering from urinary tract infections (UTI) got the final report probably 1 day earlier, saving valuable time and reducing the length of stay for inpatients. It is noteworthy that this reduction was not accompanied by a loss of sensitivity. Automated handling of urine specimens and optimized incubation periods even resulted in higher detection rates for fastidious organisms, e.g., *Alloscardovia* spp. or *Aerococcus* spp., when analyzing huge sample numbers (6).

Both automated systems meanwhile include software algorithms that can interpret digital images by themselves and segregate positive and negative cultures. The BD Kiestra has a full *in vitro* diagnostic (IVD) license in Europe, while its FDA approval is still pending (expected for summer 2021), therefore, further reducing the technician’s workload. An interesting approach combines chromogenic media with artificial intelligence algorithms (AIA). These enable the device beyond the growth/no growth decision to identify specific species (in urine mainly Escherichia coli) or detect multiple pathogens (7). In the era of the global threat by increasing antimicrobial resistance, infection control is a focal point of clinical microbiology labs. The amount of surveillance cultures sent into the labs has increased dramatically over the last decade. As these specimens contain mainly nasal or rectal swabs, these can be handled efficiently on automated systems. Reducing the TAT will prevent the spread of multiresistant bugs and avoid nosocomial transmission. For methicillin-resistant Staphylococcus aureus (MRSA), it was shown that the time to report in a specific hospital could be reduced from 48 to 24 h (8). Therefore, the period of risk to spread the MRSA from the colonized patient to others could be cut down to a half. If the digital imaging of chromogenic plates is supported by artificial intelligence algorithms, even an automated scoring can be integrated in the workflow for MRSA (9) as well as for vancomycin-resistant enterococci (VRE) (10). The latter paper already calculated economic savings for the implementation of this fully automated, resulting in a cost reduction of half a million U.S. dollars per year in the specific setup.

Culbreath et al. in this issue calculated the economic benefits of the implementation of total lab automation in four different-sized labs. The productivity in the lab increased up to 90%, while the cost per specimen could be reduced by up to 47%. All benefits together resulted in annual laboratory savings of up to $1.2 million (11). This cost savings for the labs and the hospitals was accompanied also by benefits for the patients. A median reduction of TAT of 14 h was observed, which means a shortened time to specific diagnosis for the patient. For infection control in the hospitals, a faster detection of multiresistant bacteria in surveillance cultures will reduce the risk of nosocomial transmission.

The study of Culbreath et al. is the first large scale study in North America that provides an excellent elaboration of the efficiencies and cost-savings achievable by implementation of full laboratory automation in the bacteriology laboratory. The multiple benefits relating to full-time equivalents (FTE), productivity, specimen cost, and TAT will allow laboratories to provide high-quality results in the midst of declining resources and support. Incorporation of tools, such as artificial intelligence with interpretative culture algorithms, together with future improvements of automated release of negative routine and chromogenic culture results will continue to provide the microbiology community with much needed efficiencies (11).

As pointed out by Culbreath and colleagues, negative samples with no growth can be worked off already fully automated. In the near future, we also will be able to handle and process positive samples this way. The BD Kiestra IdentifA and the Copan Colibri can pick selected colonies and prepare them for identification by mass spectrometry. In addition, the BD SusceptA submodule can process bacterial suspensions for antimicrobial susceptibility testing. In a very recent study, the IdentifA even outperformed the manual processing for *Enterobacterales*. The SusceptA prototype showed a very high correlation to manually prepared automated antibiotic susceptibility testing (AST) panels (12). This study supports the future perspectives discussed in the paper of Culbreath et al.

The microbiology lab staff can be exculpated at least partially from the increasing workload in the bacteriology lab. During the current pandemic, the released staff capacity may be urgently needed for the detection of severe acute respiratory syndrome coronavirus 2 (SARS-CoV-2). Especially in smaller laboratories or in labs in Europe, which predominantly are not running 24/7, these innovations will help to achieve reductions in time to report. As stated in a very recent commentary, the future of clinical microbiology is almost here (13).

Other innovations in the clinical microbiology lab could already prove the triple benefit of an earlier diagnosis of bloodstream infections in patients linked to a much earlier adjusted optimized therapy, essential savings in costs and workload for the laboratory and finally savings for the hospital by reduced length of stay in combination with significantly reduced total hospital costs. Applying mass spectrometry for the early detection of bacteria directly from positive blood culture bottles combined with a near real-time antibiotic stewardship provided this triple benefit (14). A subculture analysis of this huge study focusing on patients with bacteremia caused by Gram-negative rods even revealed a significant reduction of the inpatient mortality by this intervention (15). A comparable clinical study was not yet performed for total lab automation, although all prerequisites like shortened time to report, higher analytical sensitivity, and optimized performance were proven already. Therefore, the final goal of reduced mortality in infectious diseases by implementation of full lab automation cannot be claimed yet.

In many countries of the world, a shortage of trained laboratory staff, microbiological technicians, and/or technologists, was observed. This problem can result in understaffed lab areas, meaning delayed reports and increasing patient risk. Optimizing the productivity, e.g., by enhancing the number of specimens handled per FTE, can help to overcome this problem as shown by Culbreath and colleagues in this issue (11).

Meanwhile, many procedures in the microbiology lab were automated, including blood culture analysis, automated AST instruments, and recently, the culture workflow starting with specimen inoculation up to final imaging analysis. Microscopy is one of the oldest methods in our area of expertise. It has been performed manually for nearly 350 years since Antonius van Leuwenhoek discovered this great opportunity in 1683. Nowadays, first automated microscopy systems are installed in the labs as prototypes. As investigating slides under a microscope is laborious and time-consuming, such devices can significantly reduce the workload of the technical staff. Similar to automation, machine learning and artificial intelligence algorithms can optimize performance. Sensitivity and especially specificity of the device are trained and enhanced during daily routine by the AIA. Meanwhile, automated microscopes can detect acid-fast bacilli from respiratory samples more efficiently than manual analysis (16). This study provides a reasonable example for the use of AIA, showcased in the perspectives of the paper by Culbreath et al.

A missing link for the optimized performance of all of these exciting innovations is a middleware information technology (IT) solution, which would connect all instruments in the laboratory. It should be not only bidirectional but multidirectional, connecting the different devices in real time. Microscopic slides should be prepared and labeled in the streaking device of the lab automation and sent to the intelligent microscope, which of course must be able to scan and decipher the barcode label of the slide. Aliquots for molecular analysis also should be pipetted in the streaking device, labeled there, and sent to the PCR or sequencing unit. All of these processes in the lab should not be unidirectional, but there should be a network between all automated devices. It is a future goal that instruments from different suppliers could be connected in this network and talk to each other to deliver rapid and optimized results for the benefit of the patient.
